# Understanding patient and physician responses to various cost-sharing programs for prescription drugs in South Korea: A multilevel analysis

**DOI:** 10.3389/fpubh.2022.924992

**Published:** 2022-08-31

**Authors:** Kyung-Bok Son, Eui-Kyung Lee, Sang-Won Lee

**Affiliations:** ^1^College of Pharmacy, Hanyang University, Ansan-si, South Korea; ^2^School of Pharmacy, Sungkyunkwan University, Suwon-si, South Korea

**Keywords:** cost-sharing schemes, prescription drugs, patient response, physician response, South Korea

## Abstract

**Introduction:**

Patient and/or physician responses are a pivotal issue in designing rational cost-sharing programs under health insurance systems.

**Objectives:**

This study aims to understand patient and/or physician responses to cost-sharing programs designed for prescription drugs in South Korea.

**Methods:**

As a framework, we took advantage of a tiered cost-sharing program, including from copayment to coinsurance (threshold 1) and reduced coinsurance (threshold 2). Given the hierarchical structure of prescriptions nested within patients, we utilized a multilevel analysis to assess effects of various cost-sharing programs on patient and/or physician responses using National Health Insurance claims data from 2018.

**Results:**

We found that a tiered cost-sharing program was effective in changing the behaviors of patients and/or physicians. Threshold 1 was found to be more effective than threshold 2 in changing their behaviors. At the prescription level, sensitivity to cost-sharing programs was associated with prescribed days of treatment and locations of prescription. In a similar vein, sensitivity to cost-sharing programs was associated with gender and age group of patients.

**Conclusion:**

A simplified cost-sharing program with extended intervals should be considered to rationalize cost-sharing programs. Specifically, a cost-sharing program designed for long-term prescriptions for chronic diseases together with an emphasis on cost transparency is required to better guide price-conscious decisions by patients and/or physicians.

## Introduction

Growing expenditures in healthcare are a major concern for current health sectors (1, 2). Many countries, including high-income and low- and middle-income countries, have developed cost-sharing programs to address the issue of rising expenses (3–5). Cost sharing, including copayment, coinsurance, and deductibles, refers to any kind of out-of-pocket payments that a patient remits after utilizing healthcare services (6). Economic theory suggests that cost sharing would reduce patients' demand for health services by increasing prices paid by patients at the time of health services utilization (7, 8). The true effects of cost sharing on demand for various health services, however, vary within health insurance systems (9, 10). For instance, cost-sharing tactics for prescription drugs might not be generalizable to effective cost sharing for doctors' outpatient or inpatient services. Similarly, the effects of cost sharing on demand for certain types of health services vary between countries with different health insurance settings (11, 12).

Researchers have investigated effects of cost-sharing programs on the use of prescription drugs (13, 14), healthcare utilization (15), and healthcare expenditures (16, 17). Health systems have devised or revised cost- sharing programs to rationalize healthcare utilization for prescription drugs. Increased cost sharing can reduce payers' expenditures for prescription drugs and patients' healthcare utilization (13, 14), implying a trade-off between efficient use of prescription drugs and access to prescription drugs. Due to such trade-offs, the relevance of cost-sharing programs as legitimate cost containment measures depends on how patients and/or physicians respond (18). The response of patients and/or physicians to cost sharing is a pivotal issue in designing rational cost-sharing programs under health insurance systems. However, it remains unclear which patients are sensitive to changes in cost sharing and under what conditions cost sharing effectively works.

This study aims to understand patient and/or physician responses to cost-sharing programs designed for prescription drugs in South Korea. We utilized a tiered cost-sharing program in performing multilevel analysis to estimate the effects of cost-sharing programs on patient and/or physician responses. In analyzing the effects of various cost-sharing programs on patient and/or physician responses, this study elucidates prescription-level and patient-level determinants of sensitivity to cost-sharing programs.

### Cost sharing for prescription drugs in South Korea

South Korea has achieved universal health coverage through its National Health Insurance (NHI) program (19). Table 1 presents cost-sharing programs designed for prescription drugs in South Korea (20). Age of patient and total pharmaceutical expenditure per prescription are determinants of which types of cost sharing patients are responsible for paying. Total pharmaceutical expenditure is composed of pharmaceutical cost (or the sum of the reimbursed price) and the dispensing fee. Under the basic program, a patient pays 30% of a total pharmaceutical expenditure (i.e., coinsurance at 30%). The Korean government has introduced reduced cost-sharing programs for geriatric and pediatric patients. Pediatric patients under 6 years old pay 21% of the total pharmaceutical expenditure (equaling a discount of 30% in comparison to the basic program). Tiered cost sharing is applicable to geriatric patients aged 65 years or above. For geriatric patients in the first bracket, copayment is one thousand Korean Won (0.77 United States dollar, USD). Coinsurance at 20 and 30% are then applied to the second and third brackets, respectively. Finally, cost sharing for all patients is rounded down to 100 KRW. If the total pharmaceutical expenditure per prescription is 9.6 thousand KRW (for geriatric patients in the first bracket), then the patient will pay one thousand KRW. If the total pharmaceutical expenditure per prescription is 10.4 thousand KRW (for geriatric patients in the second bracket), then the patient will pay two thousand KRW. Note that the amount owed by patients in these cost-sharing cases increased from one thousand KRW to two thousand KRW.

**Table 1:** Cost-sharing programs designed for prescription drugs in South Korea.

**Type**	**Age of patient (years)**	**Total pharmaceutical expenditure per prescription**	Copay or coinsurance^*^
Non-tiered	Pediatric patients 5 and under	-	Coinsurance at 21%
	Patients between 6 and 64	-	Coinsurance at 30%
Tiered	Geriatric patients 65 and above	Equal to or <10 thousand KRW	Copayment of one thousand KRW
		Between 10 and 12 thousand KRW	Coinsurance at 20%
		Above 12 thousand KRW	Coinsurance at 30%

^*^Cost sharing for all patients is rounded down to 100 KRW.

## Methods

### Study design

**Figure 1:**
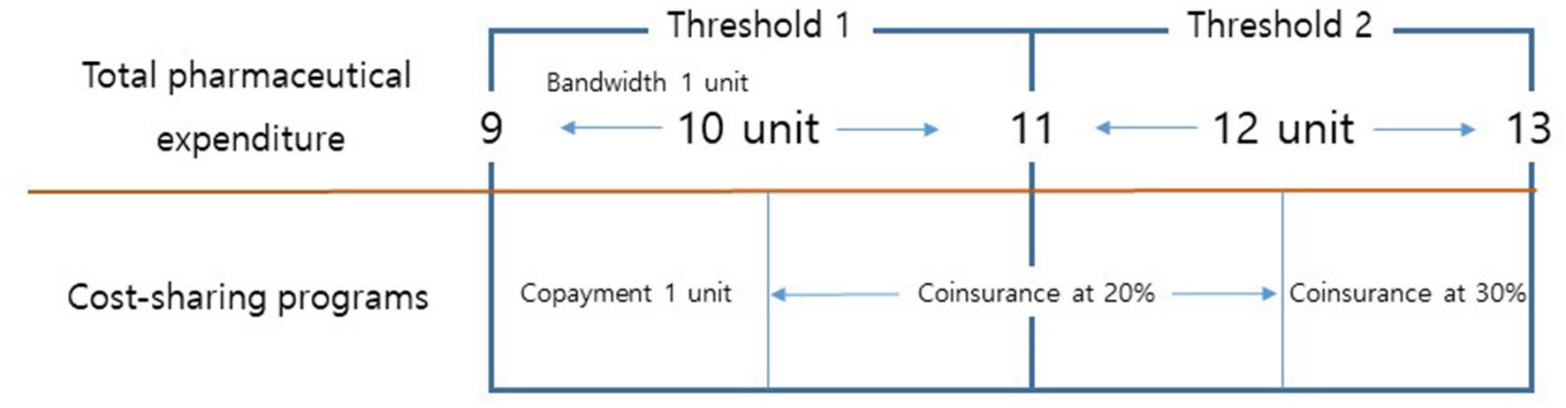
Total pharmaceutical expenditure and cost-sharing programs for prescriptions for geriatric patients. Note: One unit indicates 1,000 KRW.

Figure 1 depicts our study design. This study is interested in the responses of patients and/or physicians to various cost-sharing programs designed for prescriptions for geriatric patients. Given the three brackets for geriatric patients, our study design utilizes two thresholds—namely, 10 thousand KRW (7.69 USD) and 12 thousand KRW (9.23 USD). Geriatric patients below the first threshold were assigned to copayments of one thousand KRW (0.77 USD). Geriatric patients above the first threshold and below the second threshold were assigned to coinsurance at a rate of 20%. Geriatric patients above the second threshold were assigned to coinsurance at a rate of 30%. Any prescription below a pharmaceutical expenditure threshold was defined as being sensitive to cost-sharing programs, while any prescription above a pharmaceutical expenditure threshold was defined as being non-sensitive to cost-sharing programs.

### Data sources and materials

The Health Insurance Review and Assessment Service (HIRA) reviews all claims for reimbursement under the NHI program. Claims data include information on patients, healthcare services, prescriptions for outpatients, and healthcare institutions. Annually, the HIRA randomly selects 3% (1.4 million individuals) of the total population, designated as the National Patient Sample (HIRA-NPS), and releases their claims data for research and policy purposes. We used the 2018 HIRA-NPS for the present study (21).

Claims for prescription drugs substantially vary depending on the characteristics of patients, healthcare institutions, and health insurances. Given these factors, we selected eligible prescriptions for the analysis. First, we included prescriptions intended for patients aged 60 years and above. In South Korea, reduced cost-sharing programs are applied for geriatric patients aged 65 years and above. Patients between 60 and 64 years old were selected as the control group. The remaining patients aged 65 years and above were selected as the case group. Second, we included prescriptions prescribed at primary healthcare institutions with a bandwidth of one thousand KRW. Specifically, we chose prescriptions that cost between 9 thousand KRW and 11 thousand KRW for the threshold of 10 thousand KRW. Along these lines, we chose prescriptions that cost between 11 thousand KRW and 13 thousand KRW for the 12 thousand KRW threshold. Prescriptions within the bandwidth were assumed to be identical in terms of types of prescribed drugs. Third, we included prescriptions intended for patients who were members of the NHI. Non-NHI members are eligible for reduced cost-sharing programs. In particular, members of the Medical Aid program for low-income households were excluded from our analysis.

#### Variables

The dependent variable of this study was being sensitive to cost-sharing programs. The variable was identified under two assumptions. First, prescriptions on either side of a threshold are very similar to each other. Note that, we used thresholds with bandwidths to identify eligible prescriptions. For threshold 1, we identified prescriptions costing between 9 thousand KRW and 11 thousand KRW (corresponding to the threshold of 10 thousand KRW with a bandwidth of 1 thousand KRW). We assumed prescriptions in this group were identical in terms of types of prescribed drugs. Note that prescriptions costing between 9 thousand KRW and 11 thousand KRW were not high-tier enough for physicians to write a prescription with high-cost drugs, to establish longer prescribed days of treatment, or to increase the number of prescribed drugs. Second, patients and/or physicians could manipulate the total pharmaceutical expenditure of a prescription to gain eligibility for reduced cost-sharing programs. Physicians could prescribe a limited number of drugs or low-priced generic drugs instead of expensive drugs to adjust total pharmaceutical expenditures under the given threshold.

#### Model specification

We aim to understand patient and/or physician responses to cost-sharing programs designed for prescription drugs in South Korea. Prescriptions are main materials for this study. Given the hierarchical structure of prescriptions nested within patients, we utilized a two-level model. The multilevel model represented the outcome for prescription *i* within patient *j*. Being sensitive to cost-sharing programs at the prescription level was the outcome for the model. First, a separated prescription-level regression model was defined for each patient. Then, prescription-level coefficients were modeled as a function of patient-level predictors.

Designating the prescription level as level 1, we used number of prescribed days of treatment (prescribed days) and location of prescription as independent variables of interest. Location of prescription was categorized into three groups—metropolitan, urban, and rural areas. Metropolitan areas were Seoul, Gyeonggi-do, and Incheon. Urban areas included Busan, Daejeon, Daegu, Gwangju, Sejong, and Ulsan. Rural areas included remaining regions such as Chungcheong-do, Gangwon-do, Gyeongsang-do, Jeonla-do, and Jeju-do. At level 2, we used gender and age of patients as independent variables. In the 2018 HIRA-NPS, age of patients is grouped in 16 strata at 5-year intervals. Thus, we used age as a categorical variable. Numerical variables at level 1 were group-centered, while numerical variables at level 2 were grand-mean centered. However, categorical variables at level 1 and level 2 were not centered.

We used two types of multilevel model. Model I was designed to analyze effects of various cost-sharing programs on patient and/or physician responses. We included case and control groups in this model. Patients between 60 and 64 years of age were assigned to the control group and patients between 65 and 69 years of age were assigned to the case group. Model II was designed to understand how, for whom, and under what conditions cost-sharing programs could work among geriatric patients. For this purpose, we included case groups with three different age strata including between 65 and 69 years old, between 70 and 74 years old, and 75 years old and above.

For Model I and Model II, we applied a null model (Null), a model with variables at prescription levels (A1 and A2), and a model with variables at prescription and patient levels (B). A null model was estimated to investigate whether differences in being sensitive could be found at the prescription level and at the patient level. The difference in deviance between models, in particular a chi-squared test, was used to investigate whether each model significantly fitted the data better than the null model. In a similar vein, we used AIC and BIC to select the final model. Data management and analysis were performed using R statistical software (version 3.6.3). The “lme4” and “lmerTest” packages was used to perform a multilevel logistic regression. Significance was set at a *p*-value of < 0.05.

### Sensitivity analysis

We performed several sensitivity analyses. The bandwidth to identify eligible prescriptions was 1 thousand KRW for our main analysis. Additionally, we used 0.5 thousand KRW bandwidths for sensitivity analyses. Results for sensitivity analyses are presented in the study Appendices.

## Results

### Descriptive statistics

We categorized prescriptions and patients into sensitive and non-sensitive groups according to whether sensitivity to cost-sharing programs was detected. For prescriptions, designation as sensitive referred to prescriptions that were below the threshold. For patient categorization, patients whose portions of sensitive prescriptions out of all eligible prescriptions were above two thirds were categorized as sensitive. Note that eligible prescriptions were prescriptions for which total pharmaceutical expenditures ranged between the threshold minus one thousand KRW bandwidth and the threshold plus one thousand KRW bandwidth.

Table 2 presents two level characteristics of variables for Model I. For threshold 1, 147,681 (57%) prescriptions out of 257,880 prescriptions were assigned to the sensitive group. At level 2, 36,535 (42%) patients out of 88,206 patients were assigned to the sensitive group. When patients were divided into control and case 1 groups, the portion of sensitivity to cost-sharing programs was higher in the case 1 group than in the control group (45 vs. 40%). For threshold 1, we found that all variables at both levels presented significant differences in their distributions between sensitive and non-sensitive groups. For threshold 2, the variables of location of prescription and age of patient did not present significant differences between sensitive and non-sensitive groups.

**Table 2:** Two level characteristics of variables for Model I.

	**Threshold 1**					**Threshold 2**				
	**Non-sensitive**		**Sensitive**		***p*-value**	**Non-sensitive**		**Sensitive**		***p*-value**
**Level 1 (Prescription-level)**	*N* = 110,199		*N* = 147,681			*N* = 81,530		*N* = 96,697		
Prescribed days (mean, SD)	2.01 (3.63)		1.73 (3.36)		<0.0001	4.33 (2.34)		4.05 (2.40)		<0.0001
Location					<0.0001					0.1248
Metropolitan	49,617	(45%)	60,891	(55%)		37,104	(46%)	43,597	(54%)	
Urban areas	24,388	(41%)	35,296	(59%)		17,858	(45%)	21,509	(55%)	
Rural areas	36,194	(41%)	51,494	(59%)		26,568	(46%)	31,591	(54%)	
**Level 2 (Patient-level)**	*N* = 50,671		*N* = 37,535			*N* = 42,612		*N* = 32,601		
Gender					0.0019					<0.0001
Male	21,580	(57%)	16,378	(43%)		17,936	(56%)	14,257	(44%)	
Female	29,091	(58%)	21,157	(42%)		24,676	(57%)	18,344	(43%)	
Age, years					<0.0001					0.7150
Control (60–64)	29,363	(60%)	19,912	(40%)		24,395	(57%)	18,708	(43%)	
Case 1 (65–69)	21,308	(55%)	17,623	(45%)		18,217	(57%)	13,893	(43%)	

Table 3 presents two level characteristics of variables for Model II. For threshold 1, 286,463 (64%) prescriptions out of 446,951 prescriptions were assigned to the sensitive group. At level 2, 54,905 (47%) patients out of 116,035 patients were assigned to the sensitive group. When patients were grouped into case 1, case 2, and case 3, the portion of sensitivity to cost sharing was shown to increase by group in age order (45% for case 1, 47% for case 2, and 49% for case 3). We found that all variables, excluding patient gender, showed significant differences in distribution between sensitive and non-sensitive groups.

**Table 3:** Two level characteristics of variables for Model II.

	**Threshold 1**					**Threshold 2**				
	**Non-sensitive**		**Sensitive**		***p*-value**	**Non-sensitive**		**Sensitive**		***p*-value**
**Level 1 (Prescription-level)**	*N* = 160,488		*N* = 286,463			*N* = 109,797		*N* = 137,017		
Prescribed days (mean, SD)	3.63 (2.21)		3.43(1.91)		<0.0001	4.46 (2.59)		4.17(2.71)		<0.0001
Location					<0.0001					<0.0001
Metropolitan	67,874	(40%)	103,210	(60%)		47,349	(45%)	57,694	(55%)	
Urban areas	32,584	(34%)	63,447	(66%)		21,348	(43%)	28,062	(57%)	
Rural areas	60,030	(33%)	119,806	(67%)		41,100	(44%)	51,261	(56%)	
**Level 2 (Patient-level)**	*N* = 61,130		*N* = 54,905			*N* = 52,760		*N* = 41,406		
Gender					0.1013					0.0490
Male	25,139	(53%)	22,318	(47%)		21,365	(56%)	17,031	(44%)	
Female	35,991	(52%)	32,587	(48%)		31,395	(56%)	24,375	(44%)	
Age, years					<0.0001					0.0073
Case 1 (65–69)	21,308	(55%)	17,623	(45%)		18,217	(57%)	13,893	(43%)	
Case 2 (70–74)	16,090	(53%)	14,328	(47%)		13,724	(56%)	10,939	(44%)	
Case 3 (75-)	23,732	(51%)	22,954	(49%)		20,819	(56%)	16,574	(44%)	

### Multilevel modeling

Tables 4, 5 present estimated odds ratios for being sensitive to cost-sharing programs in Model I and Model II, respectively. Model I analyzed the effects of cost-sharing programs on patient and/or physician responses by comparing the case 1 group (comprising patients aged 65–69 years) to the control group (patients aged 60–64 years). The case 1 variable (with the control group as reference) presented a significant association with price sensitivity in threshold 1 [adjusted odds ratio (AOR): 1.35] and threshold 2 (AOR: 1.03). For threshold 1, the following predictors also showed significant associations with being price sensitive: prescribed days of treatment (also known as prescribed days), urban and rural areas (with metropolitan areas as reference), and being female (with male as reference). In a similar vein, prescribed days and urban areas showed significant associations with price sensitivity in threshold 2. In threshold 2, however, the variables of rural areas and being female did not show significant associations with price sensitivity.

**Table 4:** Estimated odds ratios for being sensitive to cost-sharing programs in Model I.

	**Threshold 1**				**Threshold 2**			
	**Null**	**A1**	**A2**	**B**	**Null**	**A1**	**A2**	**B**
Fixed effects								
**Level 1 (Prescription-level)**								
Intercept	1.36 ***	1.36 ***	1.24 ***	1.05 ***	1.25 ***	1.25 ***	1.24 ***	1.22 ***
Prescribed days		0.89 ***	0.89 ***	0.89 ***		0.92 ***	0.92 ***	0.92 ***
Urban areas (ref: metropolitan)			1.22 ***	1.21 ***			1.05 *	1.05 *
Rural areas (ref: metropolitan)			1.17 ***	1.16 ***			1.00	1.00
**Level 2 (Patient-level)**								
Female (ref: male)				1.05 ***				1.00
Case 1 (ref: control)				1.35 ***				1.03 *
Random effects								
Random intercept	1.271	1.288	1.280	1.252	1.164	1.176	1.176	1.176
Intraclass correlation coefficient	0.2787	0.2814	0.2801	0.2757	0.2613	0.2633	0.2633	0.2633
Goodness-of-fit								
AIC	331530.2	330638.5	330462.2	329915.1	235095.4	234684.0	234681.3	234680.4
BIC	331551.1	330669.9	330514.5	329988.4	235115.6	234714.3	234731.7	234751.0
χ^2^		893.7	1074.0	1625.1		413.3	420.13	425.02
df		1	3	5		1	3	5
*p*		<0.0001	<0.0001	<0.0001		<0.0001	<0.0001	<0.0001
Reference		Null	Null	Null		Null	Null	Null

*p < 0.05, **p < 0.001, ***p < 0.0001.

**Table 5:** Estimated odds ratios for being sensitive to cost-sharing programs in Model II.

	**Threshold 1**				**Threshold 2**			
	**Null**	**A1**	**A2**	**B**	**Null**	**A1**	**A2**	**B**
Fixed effects								
A. Level 1 (Prescription-level)								
Intercept	1.83 ***	1.83 ***	1.54 ***	1.33 ***	1.33 ***	1.33 ***	1.30 ***	1.25 ***
Prescribed days		0.93 ***	0.93 ***	0.93 ***		0.93 ***	0.93 ***	0.93 ***
Urban areas (ref: metropolitan)			1.36 ***	1.36 ***			1.09 ***	1.09 ***
Rural areas (ref: metropolitan)			1.34 ***	1.32 ***			1.03	1.03
B. Level 2 (Patient-level)								
Female (ref: male)				1.06 ***				1.01
Case 2 (ref: Case 1)				1.12 ***				1.06 ***
Case 3 (ref: Case 1)				1.22 ***				1.05 ***
Random effects								
Random intercept	1.755	1.766	1.745	1.733	1.425	1.438	1.437	1.436
Intraclass correlation coefficient	0.3479	0.3493	0.3466	0.3450	0.3022	0.3042	0.3040	0.3039
Goodness-of-fit								
AIC	526472.2	525660.9	525038.9	524812.8	318962.0	318423.3	318404.7	318394.9
BIC	526494.2	525693.9	525094.0	524900.9	318982.9	318454.6	318456.8	318478.2
χ^2^		813.27	1439.2	1671.3		540.71	563.29	579.13
df		1	3	6		1	3	6
*p*		<0.0001	<0.0001	<0.0001		<0.0001	<0.0001	<0.0001
Reference		Null	Null	Null		Null	Null	Null

*p < 0.05, **p < 0.001, ***p < 0.0001.

Model II determined predictors of price sensitivity to cost-sharing programs among geriatric patients. Case 2 and case 3 variables (with case 1 as reference) presented significant associations with the outcome variable for threshold 1 (AOR: 1.12 and AOR: 1.22, respectively). Case 2 and case 3 variables presented consistent results for threshold 2 (AOR: 1.06 and AOR: 1.05, respectively). Similar to findings from Model I, prescribed days, urban and rural areas (with metropolitan areas as reference), and being female showed significant associations with the outcome variable for threshold 1. Prescribed days and urban areas (with metropolitan areas as reference) also showed significant associations with the outcome variable for threshold 2.

### Sensitivity analysis

Appendices 1, 2 present sensitivity analyses for Model I and Model II, respectively. We applied 0.5 thousand KRW bandwidths for sensitivity analysis and found results that are consistent with the results of our main analysis except for two things. First, case 1 (with the control group as reference) was not a significant factor of being price sensitive for threshold 2 in Model I. Second, rural areas (with urban areas as reference) were a significant factor of being price sensitive for threshold 2 in Model II.

## Discussion

Cost sharing is a topic of much debate in health sectors. Increased cost sharing can reduce patients' utilization of healthcare. However, the responses of patients and/or physicians to cost-sharing programs when patients do utilize services remain unclear. This study is notable for analyzing the effects of various cost-sharing programs on patient and/or physician responses with taking advantage of a tiered cost-sharing program and a multilevel analysis. Findings from this study shed light on designing rational cost-sharing programs for prescription drugs.

### Interesting findings

**Figure 2:**
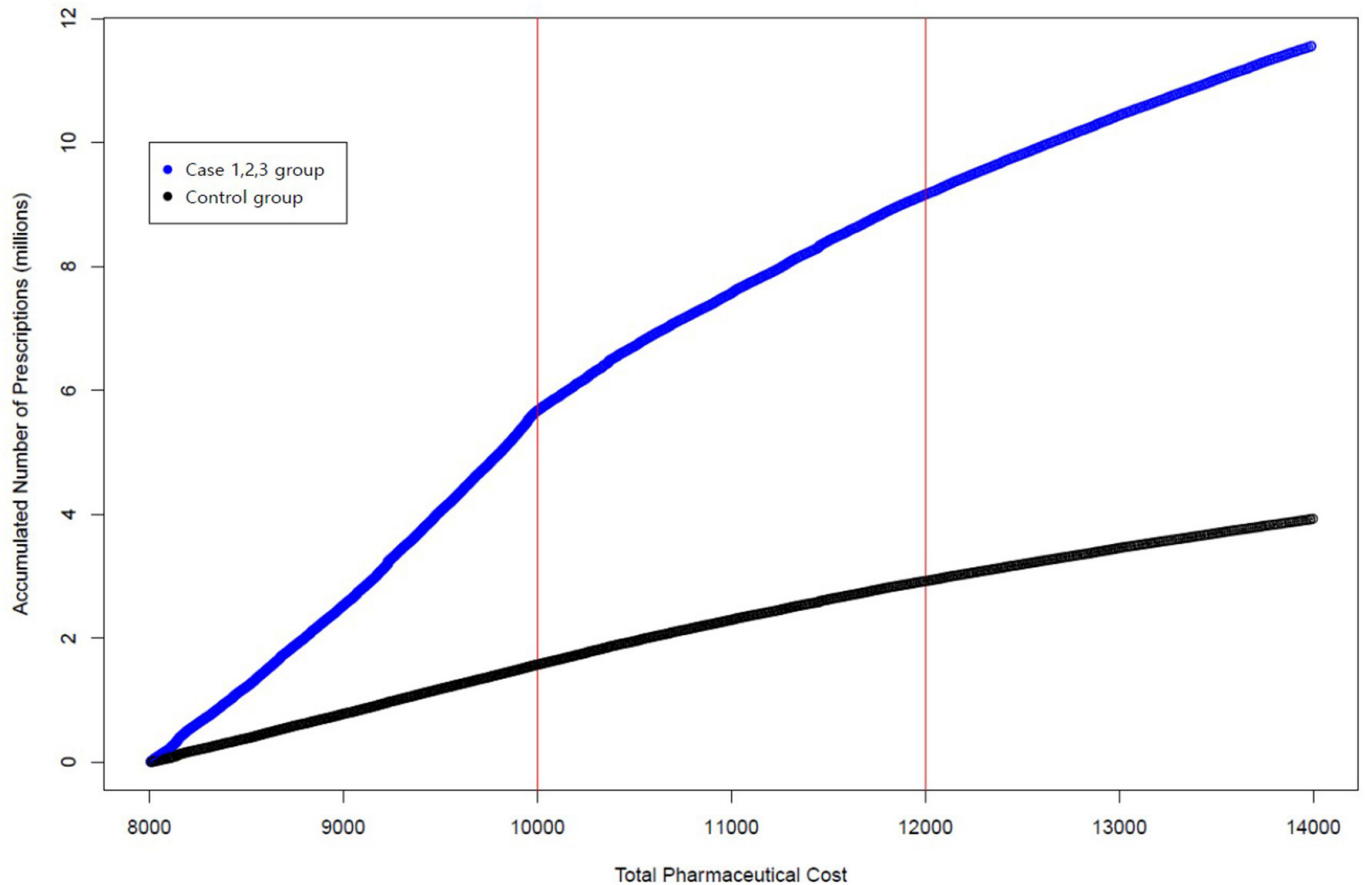
Accumulated prescriptions for the control and case groups.

We conducted multilevel analysis to estimate the effects of various cost-sharing programs designed for prescription drugs on patient and/or physician responses. This study reveals interesting findings. A tiered cost-sharing program —copayment and reduced coinsurance—were found to be effective in changing the behaviors of patients and/or physicians. Physicians were more likely to prescribe treatments that cost below a given threshold for patients who were eligible for reduced cost-sharing programs. However, the sizes of effects differed between two thresholds. Threshold 1 specified the effects of increased cost sharing from a copayment of 1 thousand KRW to 20% coinsurance, while threshold 2 reflected the effects of increased coinsurance from 20 to 30%. In a multilevel logistic regression model, threshold 1 was found to be more effective than threshold 2 in changing the behaviors of patients and/or physicians. This finding is in line with the results of our descriptive analysis. Figure 2 presents accumulated prescriptions for the control and case groups. The horizontal axis indicates total pharmaceutical expenditures, and the vertical axis indicates the accumulated number of prescriptions. The two red vertical lines indicate thresholds 1 and 2, respectively. In Figure 2, we see that the slope of the curve on different sides of the two thresholds is not noticeably changed for the control group. However, the slope of the curve on different sides of the two thresholds is notably changed for the case group. Furthermore, changes in slope among the case group were marginal for threshold 2 in comparison to changes in slope among the case group for threshold 1, thereby implying that threshold 2 was not quite effective in changing the behaviors of patients and/or physicians.

Studies have documented that increased cost sharing can reduce healthcare utilization, particularly for patients with low income and for female patients (22, 23). Regardless, not much empirical research has analyzed the effects of cost-sharing programs in terms of the behavior of patients and physicians. We could not measure the effects of cost-sharing programs on total healthcare utilization. We could, however, measure the responses of patients and/or physicians to various cost-sharing programs when patients were prescribed treatments. We found that prescribed days of treatment and location of prescription at the prescription level and that gender and age group at the patient level were associated with sensitivity to cost-sharing programs. It is realistic to assume that geriatric patients aged 70 and above, as well as female geriatric patients, are more likely to be price sensitive than patients between 65 and 69 years old and male geriatric patients. At the prescription level, longer courses of treatment (prescribed days) were more likely to show non-sensitivity to cost-sharing programs. This negative association may be explained by the structure of total pharmaceutical expenditure, which is composed of pharmaceutical cost and dispensing fee. The former is associated with the sum of the reimbursed price of prescribed drugs and the latter is related to prescribed days of treatment. As prescribed days of treatment increase, so does the sum of the reimbursed price and its dispensing fee.

### Designing rational cost-sharing programs

Findings from this study stand to provide implications for designing rational cost-sharing programs. Cost sharing was originally designed to constrain rising healthcare utilization and expenditures. Conceptually, cost sharing seemed to provide financial incentives for patients to raise their awareness of healthcare costs. Increased awareness of healthcare costs was supposed to guide well-informed decisions on the part of patients in utilizing healthcare services. However, researchers have analyzed a common problem behind cost-sharing programs—namely, an overly complex design that is hardly understood by patients (24–27). Neither patients nor physicians are able to predict expenses under the complexities of cost sharing. Accordingly, practitioners and patients alike are less likely to make well-informed or price-conscious decisions.

In line with previous findings, we discovered that a tiered cost-sharing program with short intervals might not be quite effective in terms of patient and/or physician behaviors. Cost-sharing programs in South Korea have two thresholds − 10 thousand KRW and 12 thousand KRW. While these two thresholds are herein shown to be effective in changing patient and/or physician behaviors, the effectiveness of the second threshold turned out to be marginal. This is partially explained by the complexity of the cost-sharing program. First, the program has a marginal interval between the two thresholds, which is two thousand KRW. Second, the program utilizes two types of cost-sharing payments for three brackets of patients. The first bracket uses copayment (1 thousand KRW), and the second and third brackets use 20 and 30% coinsurance. Given these details, we suggest a simplified cost-sharing program with extended intervals.

Next, we suggest a cost-sharing program for long-term prescriptions for chronic diseases, including hypertension, diabetes, and hypercholesterolemia. The two thresholds were mainly designed for prescriptions with short prescribed days of treatment, indicating that another threshold could be introduced for prescriptions with longer prescribed days of treatment. If a higher threshold could be introduced for prescription-based treatments for chronic diseases, then patients and/or physicians might prefer low-priced generics to high-priced brand-name drugs in order to keep the prescription under the threshold. Along these lines, South Korea has implemented differential cost-sharing programs for doctors' consultation fees to increase utilization of primary healthcare institutions for the management of chronic diseases (28). The schemes utilize increased coinsurance rates for patients who visit tertiary and secondary hospitals for the management of chronic disease. The programs have been effective in guiding patients with hypertension to visit primary healthcare institutions (28–30). Creating systems under which physicians act as perfect agents of patients who prefer low-priced generics to high-priced generics or brand-name drugs is necessary for long-term prescriptions for chronic diseases. For instance, the association of being prescribed low-priced drugs with the existence of usual source of care was reported for patients with chronic diseases in South Korea (31).

Finally, we emphasize the importance of cost transparency in designing cost sharing for prescription drugs. Economic theory suggests that if a patient knows the price of a prescription drug, he or she will make a well-informed decision by avoiding high-priced options (32). It is difficult for patients, however, to know the sum of reimbursement prices of prescribed drugs upon receiving a prescription (33). Furthermore, dispensing fees do not increase proportionally as the prescribed days of treatment increase. Thus, neither patients nor physicians can calculate total pharmaceutical expenditures and cost sharing that a patient must pay. Under these circumstances, cost transparency has the potential to guide cost sensitive patients and/or physicians to keep the pharmaceutical expenditures of prescriptions under a threshold. Cost transparency is deemed as an important component of health insurance to lower the cost of prescription drugs in the United States. Thus, 191 legislations were introduced to improve cost transparency at the states level (34).

### Study limitations

This study has several limitations. First, this study assumes that prescriptions on different sides of thresholds are very similar to each other and provides bandwidths of 0.5 and 1 thousand KRW to validate our assumption. However, prescriptions on different sides of a threshold might present different characteristics. Second, this study analyzes the responses of patients and/or physicians against various cost-sharing programs in cases where patient treatment requires prescription. Thus, we cannot measure the effects of cost-sharing programs on total healthcare utilization. Third, this study utilizes being sensitive to cost-sharing program as the dependent variable to capture patient and/or physician response. However, further research is needed to investigate the mechanism through which the changes in patient and/or physician response occur. Fourth, this study notes the importance of cost sensitivity in measuring patient and/or physician responses. Nevertheless, patients and/or physicians might respond more meaningfully to the quality of care than to the cost of care. For prescription drugs, patients and/or physicians might prefer high-price brand-name drugs instead of low-price generic drugs. Finally, this study analyzes patient and/or physician responses to cost-sharing programs designed for prescription drugs in South Korea. Finding from this study could not be generalized to other health insurance systems with different contexts.

## Conclusion

The response of patients and/or physicians is a pivotal issue in designing rational cost-sharing programs under health insurance systems. We conducted multilevel analysis to estimate the effects of cost-sharing programs on patient and/or physician responses. Our analysis reveals that a tiered cost-sharing program—copayment and reduced coinsurance—are effective in changing the behaviors of patients and/or physicians. Physicians were shown to be more likely to prescribe medications at prices below the threshold for patients who were eligible for reduced cost-sharing programs. However, threshold 1 was more effective than threshold 2 in changing patient and/or physician behaviors. A simplified cost-sharing program with extended intervals should be considered to rationalize cost-sharing programs. Specifically, a cost-sharing program designed for long-term prescriptions for chronic diseases together with an emphasis on cost transparency is required to better guide price-conscious decisions by patients and/or physicians.

## Data availability statement

The data that support the findings of this study are available from HIRA but restrictions apply to the availability of these data, which were used under license for the current study, and so are not publicly available.

## Author contributions

K-BS developed the concept of the study, undertook the analysis, and wrote the manuscript. E-KL developed the concept of the study and revised the manuscript. S-WL developed the concept of the study, wrote the manuscript, and revised the manuscript. All authors contributed to the article and approved the submitted version.

## Conflict of interest

The authors declare that the research was conducted in the absence of any commercial or financial relationships that could be construed as a potential conflict of interest.

## Publisher's note

All claims expressed in this article are solely those of the authors and do not necessarily represent those of their affiliated organizations, or those of the publisher, the editors and the reviewers. Any product that may be evaluated in this article, or claim that may be made by its manufacturer, is not guaranteed or endorsed by the publisher.

## Appendix

**Table A1:** Sensitivity analysis with different bandwidth for Model I.

	**Threshold 1**	**Threshold 2**
	Bandwidth 500	Bandwidth 500
	132,541 prescriptions 60,878 patients	87,825 prescriptions 48,421 patients
Fixed effects		
A. Level 1 (Prescription-level)		
Intercept	0.98	1.13 ***
Prescribed days	0.95 ***	0.97 ***
Urban areas (ref: metropolitan)	1.23 ***	1.10 ***
Rural areas (ref: metropolitan)	1.20 ***	1.04
B. Level 2 (Patient-level)		
Female (ref: male)	1.04 *	0.99
Case 1 (ref: control)	1.39 ***	1.00
Random effects		
Random intercept	1.951	1.728
Intraclass correlation coefficient	0.3723	0.3444
Goodness-of-fit		
AIC	167606.3	115038.0
BIC	167674.8	115103.7
χ^2^	486.63	42.05
df	5	5
*p*	<0.0001	<0.0001
Reference	Null	Null

**p* < 0.05, ***p* < 0.001, ****p* < 0.0001.

**Table A2:** Sensitivity analysis with different bandwidth for Model II.

	**Threshold 1**	**Threshold 2**
	Bandwidth 500	Bandwidth 500
	236,546 prescriptions 84,132 patients	121,325 prescriptions 61,396 patients
Fixed effects		
A. **Level 1 (Prescription-level)**		
Intercept	1.26 ***	1.10 ***
Prescribed days	0.98 **	0.98 *
Urban areas (ref: metropolitan)	1.47 ***	1.11 ***
Rural areas (ref: metropolitan)	1.42 ***	1.09 ***
B. **Level 2 (Patient-level)**		
Female (ref: male)	1.07 ***	1.01
Case 2 (ref: case 1)	1.11 ***	1.11 ***
Case 3 (ref: case 1)	1.17 ***	1.08 **
Random effects		
Random intercept	2.853	2.176
Intraclass correlation coefficient	0.4644	0.3981
Goodness-of-fit		
AIC	271424.2	155169.6
BIC	271507.2	155247.2
χ^2^	521.98	54.38
df	6	6
*p*	<0.0001	<0.0001
Reference	null	null

**p* < 0.05, **p* < 0.001, ****p* < 0.0001.
